# Impact of Physical Activity on the Incidence of Vascular Diseases in Adults with Type 2 Diabetes Mellitus

**DOI:** 10.31661/gmj.v8i0.1549

**Published:** 2019-05-20

**Authors:** Babak Pezeshki, Ehsan Bahramali, Amir Ansari, Aliasghar Karimi, Mohammad Sabet, Mojtaba Farjam, Azizallah Dehghan

**Affiliations:** ^1^Noncommunicable Diseases (NCD) Research Center, Fasa University of Medical Sciences, Fasa, Iran; ^2^Student Research Committee of Fasa University of Medical Sciences, Fasa, Iran

**Keywords:** Physical Activity, Diabetes Mellitus, Vascular Diseases, Myocardial Infarction, Stroke

## Abstract

**Background:**

Diabetes mellitus (DM) is a common metabolic disease worldwide and has many complications. Vascular events are the major complication of DM, which have an important effect on mortality and disability. Physical activity (PA) enhances the vascular function by several pathways. The aim of this study was to evaluate the relationship between PA and vascular diseases in patients with DM.

**Materials and Methods:**

This study was performed as a case-control study extracted from a prospective epidemiological research study in Iran. Patients with type 2 DM for more than six months as a case group were compared to sex- and age-matched healthy control subjects. The metabolic equivalent of task score was used to evaluate the level of PA and blood glucose, lipid profile, body mass index, overweight, dyslipidemia, glomerular filtration rate, myocardial infarction, unstable angina, and stroke.

**Results:**

Overall, 1242 patients with DM were extracted, and 2484 non-DM subjects were investigated. In the case group, 355 (28.6%) and 887 (71.4%) subjects were men and women, respectively, and 710 (28.6%) men and 1774 (71.4%) women were in the control group. The mean metabolic equivalent of task score was 30 and 40.97 in the DM and non-DM groups, respectively (P˂0.001). The frequency of myocardial infarction, stroke, and cardiac ischemia was 44 (3.5%), 37 (3%), and 267 (21.5%) in the DM group, and 54 (2.2%), 43 (1.7%), and 389 (15.7%) in the non-DM group, respectively.

**Conclusion:**

The incidence of vascular events associated with PA level in patients with DM and adherence to regular PA reduced vascular events and DM complications.

## Introduction

Diabetes mellitus (DM) is a common metabolic disease as a result of hyperglycemia, and the basis of the pathogenic process of this disorder is classified as type 2 or type 1 DM [1]. DM affected more than 415 million people around the world in 2017 and is estimated to affect more than 640 million individuals by 2040 [2]. Patients with DM are greatly exposed to macrovascular events such as cardiovascular diseases (CVDs) or stroke, which have important effects on the mortality and disability rate due to DM [3]. Glycemic control reduces macrovascular events in patients with type 2 DM [4, 5] and hyperglycemia, which is shown by hemoglobin A1c (HbA1c) level to be a more important risk factor for CVDs compared to lipid profile, blood pressure, and smoking. Therefore, the reduction of HbA1c level can decrease the risk of CVDs in patients with DM [6]. Exercise and physical activity (PA) increase insulin sensitivity in muscles; thus, the muscle glucose uptake is enhanced, and the muscle glycogen repletion is linked with the duration of PA [7-9]. Moreover, regular PA is likely optimal to enhanced insulin action and secretion due to increase in muscle oxidative capacity, capillary density, and lipid metabolism [7, 10]. On the other hand, increase of PA is associated with HbA1c reduction and regular exercise more than two and a half hour per week significantly decreases HbA1c level as an index to blood glucose control [3, 11-13]. Macrovascular diseases can develop in diabetic patients if they do not have suitable glycemic control [14]. Although regular PA can improve the CVD risk [11] and decrease the mortality rate in patients with DM due to improving glycemic control [3, 11, 12], the incidence of macrovascular diseases is not clear in patients with DM. The aim of this cross-sectional study was to investigate the influence of PA on glycemic control and macrovascular diseases in the national cohort of the Research Deputy of the Ministry of Health, Treatment, and Medical Education, which considers factors affecting non-communicable diseases in the Iranian population.

## Materials and Methods

In this objective case-control study, patients with type 2 DM were enrolled, who met the criteria of DM published by the American Diabetes Association (ADA) [3]. The diagnostic criteria for DM that were provided by ADA are fasting blood glucose (FBS) ≥126 mg/dL (7.0 mmol/L), 2-hour plasma glucose ≥200 mg/dL (11.1 mmol/L), HbA1C ≥6.5% (48 mmol/mol) or random plasma glucose ≥200 mg/dL (11.1 mmol/L). Patients who met these criteria were included in the DM group. On the other hand, the ratio of participants in the case and control groups was 1:2, and age and sex were matched among them. The data of the participants were provided by the Fasa cohort study conducted by the Fasa University of Medical Sciences in Sheshdeh, Fasa, Fars Province, Iran, under the supervision of a prospective epidemiological research study in Iran (PERSIAN) [15, 16]. All the participants were suffering from type 2 DM more than six months and were using medication to control the disease. PA level was measured with the metabolic equivalent of task (MET) score, as the continuous indicator of PA per week/minutes. This is a tool to access the energy rate used per week [17]. The Persian questions about PA were previously validated [18] according to patients’ self-report about their MET that was categorized in quartile deviation for each group. Increase of the quartile number shows that subjects have performed more PA. The demographic data and medical records of the patients were extracted from the database of PERSIAN. Hypertension is defined as systolic blood pressure more than 140 mmHg or diastolic blood pressure more than 90 mmHg. Moreover, hypertension associated with a new or progressive target organ dysfunction is called malignant hypertension [1, 19]. Lipid profile, FBS, and creatinine were measured in the PERSIAN laboratory. Moreover, body mass index (BMI), overweight, dyslipidemia, diabetic ketoacidosis, glomerular filtration rate, myocardial infarction (MI), unstable angina, and stroke were defined as current guidelines [1, 3]. Frequency, mean, and standard deviation (SD) were used for the descriptive analysis of quantitative variables. Demographic characteristics and clinical finding were shown as mean±SD, frequency (percentage), and the number of events. The chi-square test was used for dichotomous variables to compare the groups, and the ANOVA method was applied to correct P-values for multiple comparisons. A two-sided P-value less than 0.05 was considered significant. All the data were analyzed using the SPSS Statistics® software version 19 (SPSS Inc., Chicago, Illinois, USA).

## Results

Among the 9964 subjects registered at the Fasa branch of PERSIAN, 1242 patients with DM were extracted, and 2484 non-DM subjects were selected for the sex- and age-matched control group. There were 355 (28.6%) men and 887 (71.4%) women in the DM group with the average of age being above 53 years. In the non-diabetic group, the number of men and women was similar to that in the DM group with the ratio 1:2 and the mean age of 54 years, which was not significantly different. Other demographic data are shown in Table-1. The mean of the MET score was 30 in the DM group, whereas it was nearly 50 in the non-diabetic group, which indicated a significant difference between the two groups (P˂0.001). Moreover, the number of subjects who had a history of stroke, MI, and cardiac ischemia was significantly different between the two groups (Table-1). Table-2 shows that the increased number of quartiles was significantly associated with renal function, blood pressure, history of MI, and cardiac ischemia in both groups. However, a history of stroke was significantly associated with the level of the MET score only in the DM group. The odds ratio of stroke in the DM patients was 1.7 from 4th quartile to 1st quartile of MET score. Furthermore, the odds ratio of MI was 1.54 from 4th quartile to 1st quartile of MET score in the DM patients( Table-3).

## Discussion

DM is a chronic metabolic disease that is not treatable and has several lethal and debilitating complications. However, these complications are preventable and decrease mortality and disability [1]. In this study, we analyzed the data of a high number of adult patients with type 2 DM to evaluate the association between the self-reported MET score and vascular diseases in patients with DM. Based on our results, the MET score in patients with DM was significantly less than that in the non-DM subjects, and most of them were inactive same the other studies on DM were demonstrated the majority of them has low PA [20]. BMI in the DM and non-DM subjects was the same in this investigation, although other studies showed significantly high BMI in patients with DM [20, 21]. On the other hand, previous studies showed exercise significantly increased the blood flow in the arteries and improved the vascular function. However, aerobic exercise significantly increased the blood flow more than the combined aerobic and resistance exercises. This means that aerobic exercise was a better way to improve the blood flow in patients with DM [22]. Moreover, exercise raises antioxidant capacity by enhancing the expression of antioxidant enzymes so that the endothelial get better function and vascular smooth muscle provides better conditions for blood flow in patients with DM [23, 24]. According to our results, improvement of renal function and blood pressure was correlated with the MET score, meaning that PA improved the vascular function due to the regulation of the blood flow. Moreover, our data indicated a significant relationship between PA and the rate of lethal vascular diseases such as MI and stroke in the DM group, as shown in previous papers, showing that PA has an important role in reducing the vascular event of DM and death [25]. This study investigated the effect of PA on vascular diseases in adult patients with type 2 DM, and other age ranges and types of DM were not investigated. The effect of PA on vascular event needs more investigation as large prospective studies and trials.

## Conclusion

PA has an important role in reducing lethal vascular diseases and death. Thus, adherence to regular PA is highly important, and physicians and nurses must inform patients about DM compilations due to low PA and urge them to perform regular PA to reduce the mortality and disability of DM.

## Conflict of Interest

None.

**Table 1:** The Demographic Variables of Both Groups

**Variables**	**DM group**	**Non-DM group**	**P-value**
**Age,y (mean±SD)**	53.61±8.81	54.01±4.22	0.069
**Sex**			
Male, n(%)	355(28.6)	710 (28.6)	1
Female, n(%)	887(71.4)	1774(71.4)	
**MET score**	30.03±8.87	40.97±10.20	<0.001
**Diabetes duration,y (mean±SD)**	3.55±4.25	-	-
FBS (mean±SD)	132.19±62.29	90.08±16.83	<0.001
Systolic blood pressure	117.42 ±19.84	115.69±19.3	0.012
Diastolic blood pressure	77.23±12.07	76.53±12.39	0.103
**Medication**			
Insulin	38(3.1)	-	-
Metformin	420(33.8)	8(0.3)	<0.001
Glibenclamide	262 (21.1)	-	-
**BMI (kg/m** ^2^ **)**	26.64±4.94	26.39±4.97	0.144
**Total cholesterol (mmol/L)**	187.65±45.39	193.08±39.79	<0.001
**LDL cholesterol (mmol/L)**	106.24±37.87	112.82±33.34	<0.001
**HDL cholesterol (mmol/L)**	50.98±15.54	53.81±16.59	<0.001
**HDL-to-LDL ratio**	0.58±0.99	0.54±0.63	<0.001
**Triglycerides (mmol/L)**	152.15±98.21	134.34±80.81	<0.001
**Smoker (%)**	229 (18.4)	288 (19.6)	0.116
**Alcohol consumption(regular)**	7 (0.6)	6 (0.2)	0.497
**Stroke**	37 (3)	43 (1.7)	0.013
**Myocardial infarction**	44 (3.5)	54 (2.2)	0.014
**Cardiac ischemic**	267 (21.5)	389 (15.7)	<0.001

**FBS:** Fasting blood glucose; **LDL:** Low-density lipoprotein; **HDL:** High-density lipoprotein; **BMI:** Body mass index

**Table 2:** The Quartiles of the MET Score and Vascular Function of Both Groups

	**Group**	**Quartile 1**	**Quartile 2**	**Quartile 3**	**Quartile 4**	**P-value**
**Creatinine**	DM	1.02±0.27	0.97±0.13	0.96±0.19	0.99±0.14	<0.001
	Non-DM	0.98±0.27	0.94±0.18	0.93±0.14	0.96±0.17	<0.001
**FBS**	DM	135.02± 62.83	132.56±62.96	132.97±62.14	127.29±61/32	0.541
	Non-DM	91.18±19.86	90.91±15.63	89.22±11.62	89.27±19.24	0.082
**Glomerular filtration rate**	DM	68.43±12.66	69.24±9.65	69.37±10.47	73.07±12.08	<0.001
	Non-DM	72.17±11.59	70.73±10.03	71.17±9.58	74.89±11.65	<0.001
**Systolic blood pressure**	DM	120.66±20.92	117.60±19.44	115.71±19.29	115.01±19.06	0.002
	Non-DM	117.45±19.96	117.76±20.17	114.65±19.22	113.64±19.04	<0.001
**Diastolic blood pressure**	DM	78.77	76.69	76.26	77.08	<0.001
	Non-DM	77.49	77.88	75.67	75.47	0.043
**Hypertension (%)**	DM	182 (53.8)	161 (46.9)	132 (43.9)	86(35.1)	<0.001
	Non-DM	166 (30.9)	209 (33.9)	188 (30.1)	147 (20.8)	0.002
**Myocardial infarction**	DM	18 (5.01)	15 (4.4)	9 (3)	2 (0.8)	0.036
	Non-DM	21 (4)	10 (1.6)	15 (2.4)	8 (1.1)	0.006
**Cardiac ischemic**	DM	89 (26.3)	81 (23.6)	54(17.9)	40(16.3)	0.008
	Non-DM	101(19.1)	100(16.3)	103 (17.7)	80 (11.5)	0.011
**Stroke**	DM	18 (5.3)	11 (3.2)	5 (1.7)	3 (1.2)	0.013
	Non-DM	14 (2.6)	12 (1.8)	7 (1)	10 (1.4)	0.159

**Table 3:** The Odds Ratio of Macrovascular Diseases in the DM Patients from 4th Quartile to 1st Quartile of MET Score.

**Diseases**	**Odds Ratio**	**S.E**	**P-value**
**Stroke**	1.72	0.17	0.002
**Myocardial infarction**	1.54	0.15	0.006
**Cardiac ischemic**	1.24	0.06	0.001
